# Draft Genome Sequence of *Aspergillus oryzae* 100-8, an Increased Acid Protease Production Strain

**DOI:** 10.1128/genomeA.00548-14

**Published:** 2014-06-05

**Authors:** Guozhong Zhao, Yunping Yao, Lihua Hou, Chunling Wang, Xiaohong Cao

**Affiliations:** a Key Laboratory of Food Nutrition and Safety (Tianjin University of Science and Technology), Ministry of Education, Tianjin, China; b State Key Laboratory of Food Science and Technology, School of Food Science and Technology, Jiangnan University, Wuxi, Jiangsu Province, China

## Abstract

*Aspergillus oryzae* is a common fungus for traditional fermentation in Asia, such as spirit, soybean paste, and soy sauce fermentation. We report the 36.7-Mbp draft genome sequence of *A. oryzae* 100-8 and compared it to the published genome sequence of *A. oryzae* 3.042.

## GENOME ANNOUNCEMENT

*Aspergillus oryzae* is a strain extensively used in fermented foods and listed as generally regarded as safe (GRAS) status (1). *A. oryzae* 100-8, a close relative of *A. oryzae* 3.042, obtained by N^+^ ion implantation mutagenesis, can secrete more acid protease than *A. oryzae* 3.042 (2). Soy sauce flavors fermented by these two strains are different.

The genome sequence of *A. oryzae* 100-8 was determined with a combined strategy of Roche 454 and Solexa paired-end and Solexa mate-paired sequencing technologies. The paired-end reads (12,874,408 reads and 2.4 gigabases) and mate-paired reads (1,134,139 reads and 226.8 megabases) were generated by the Solexa sequencer and assembled by SOAPdenovo (3). With the Newbler sequence assembler (version 2.3) (4), we performed a hybrid assembly of the 454 reads and the split fragments of contigs generated by SOAPdenovo.

The draft genome of *A. oryzae* 100-8 consists of 210 sequence contigs with a total length of 36.7 Mbp and a G+C content of 48.3%. The genome length of *A. oryzae* 100-8 was less than that of *A. oryzae* RIB40 (5). The numbers of protein-coding genes and tRNAs predicted were 11,187 and 243, respectively. The protein-coding sequence occupies 44% of the sequenced portion of the genome of strain 100-8. The complete mitochondrial genome sequence of *A. oryzae* 100-8, a circular DNA molecule of 29,192 bp with a G+C content of 26%, was determined. Comparative genomic analysis was performed with the published genome of *A. oryzae* 3.042.

According to the comparison to *A. oryzae* 3.042, most of the gene sequences of strain 100-8 are the same as those of *A. oryzae* 3.042. However, nucleotide insertions, nucleotide deletions, and single nucleotide polymorphisms (SNP) were found in some genes through the sequence alignment. These changes may explain the differences of these two strains at the gene level. The transcription factor PBP and vesicle coat complex proteins may be related to regulation of the expression of many potential acid proteases (6). The transcription factor PBP was regarded as the key enzyme to regulate the expression of the different genes. The changes of some exporter, permease, and transporter genes have effects on the transport capacity (7). Differences of the other dehydrogenase genes (such as those encoding aldehyde dehydrogenase, threonine dehydrogenase, and Zn-dependent dehydrogenase) regulate the production of different flavor precursors (8).

Comparison of the genomics of *A. oryzae* 100-8 and *A. oryzae* 3.042 will provide us with a new method to understand the mechanisms of *A. oryzae*. It also clarifies the traces of different flavors produced by *A. oryzae*.

### Nucleotide sequence accession number.

This whole-genome shotgun project has been deposited at DDBJ/EMBL/GenBank under the accession number AMCJ00000000. The version described in this paper is the first version.
